# Strategies to reduce risk perception among grocery shoppers in the US: A survey study

**DOI:** 10.1371/journal.pone.0251060

**Published:** 2021-04-28

**Authors:** Jie Li, Leslie J. Verteramo Chiu, Miguel I. Gómez, Nelson L. Bills

**Affiliations:** 1 Charles H. Dyson School of Applied Economics and Management, Cornell University, Ithaca, NY, United States of America; 2 Department of Population Medicine and Diagnostic Sciences, Cornell University, Ithaca, NY, United States of America; Universidad de Los Andes, COLOMBIA

## Abstract

During the COVID-19 lockdown in the US, many businesses were shut down temporarily. Essential businesses, most prominently grocery stores, remained open to ensure access to food and household essentials. Grocery shopping presents increased potential for COVID-19 infection because customers and store employees are in proximity to each other. This study investigated shoppers’ perceptions of COVID-19 infection risks and put them in context by comparing grocery shopping to other activities outside home, and examined whether a proactive preventive action by grocery stores influence shoppers’ perceived risk of COVID-19 infection. Our data were obtained via an anonymous online survey distributed between April 2 and 10, 2020 to grocery shoppers in New York State (the most affected by the pandemic at the time of the study) and Washington State (the first affected by the pandemic). We found significant factors associated with high levels of risk perception on grocery shoppers. We identified some effective preventive actions that grocery stores implement to alleviate anxiety and risk perception. We found that people are generally more concerned about in-store grocery shopping relative to other out-of-home activities. Findings suggest that a strict policy requiring grocery store employees to use facemasks and gloves greatly reduced shoppers’ perceived risk rating of infection of themselves by 37.5% and store employees by 51.2%. Preventive actions by customers and businesses are critical to reducing the unwitting transmission of COVID-19 as state governments prepare to reopen the economy and relax restrictions on activities outside home.

## Introduction

Since the outbreak of COVID-19 until the time of writing this article (April 8, 2021), the World Health Organization (WHO) has reported 132,730,691 confirmed cases and 2,880,726 deaths in 219 countries (WHO 2021). By late April 2020, the US had become the epicenter of COVID-19. Washington State (WA) was the first epicenter in the country, but it was surpassed by New York State (NYS) by Mid-March 2020. At the time of data collection (April 2–10, 2020), NYS had the most infections and deaths. The NYS COVID-19 death rate of 7.9% was the highest among all states in the country (WHO 2020).

Social distancing and shelter in place have been recommended and enforced to varying degrees by states across the US to reduce contact rates among individuals. Despite these efforts, complying with the official recommendations, especially if living in a densely populated area, presents major challenges. Even under shelter in place, people need to leave their homes for various reasons, not the least to purchase food and other essential supplies at grocery stores.

In most cases, grocery shopping requires being physically present at the store. Although online delivery of food has increased during the pandemic, the majority of food purchases still happen at in-store because online operators do not have the capacity to provide for curbside pickup or meet the dramatic increase in demand for at-home food deliveries; many shoppers cannot afford paying for delivery fees [1]. Proximity to store employees increases the actual and perceived risk of COVID-19 infection relative to activities, especially since evidence indicates that COVID-19 can be transmitted from asymptomatic individuals [2, 3], and by aerosols from breathing and talking [4, 5]. According to the US Center for Disease Control (CDC), a social distance of at least six feet (about 1.83 meters) is necessary to prevent the virus in the expelled droplets of an infected person to reach another individual.

The potential risk of infection when grocery shopping may not be limited to proximity to other shoppers. Store employees handle foods and may also be a potential source of infection. An infected but asymptomatic employee could contaminate the surface of food products and (or) critical places such as checkout stations. The virus can survive on hard surfaces from a few hours to a few days [6]. Store employees commonly handle grocery items while customers are shopping, further increasing shoppers’ actual and perceived risk of infection.

While the risk of contagion when grocery shopping has been established, little is known about effective strategies to mitigate the perceived risk of grocery shoppers. Identifying strategies to reduce risk perception is critical for effective communication and management of global pandemics, which in turn, help reduce mass panic, unnecessary precaution behaviors and catastrophic impacts on the economy [7–9]. Addressing this issue is critical, as the COVID-19 pandemic will affect human behavior in such necessary activities as food purchases in years to come. To fill this gap, this paper analyzes risk perception among grocery shoppers, identifies risk mitigating strategies, and sheds light on effective interventions to reduce shopper anxiety over COVID-19 infection in grocery stores. Specifically, this study addresses the following questions: Is the perceived risk of grocery shoppers high in comparison to other activities? If grocery stores implement preventive procedures to mitigate COVID-19 infection, would customers feel less anxious when grocery shopping? Do shoppers perceive that grocery stores are protecting their employees from getting infected by COVID-19? Answers to these questions can provide critical information to state governments and food retailers as the COVID-19 increase and concerns emerge for the ongoing second/third wave of the pandemic.

## Methodology

### Study design and setting

An online, anonymous survey was created and distributed via the Qualtrics Survey Platform (Qualtrics, Provo, Utah, USA). The survey (available as a supplemental document) was randomly distributed among residents in NYS and WA during April 2–10, 2020. WA was selected for this study because it was the first epicenter of COVID-19 in the US, and it had the highest COVID-19-related death rate in the country when the survey was distributed (WHO Website). The study also focused on NYS because, shortly after the outbreak, it became the epicenter of the pandemic. Residents who were at least 21 years of age and were the primary grocery shoppers in the household were invited to participate in the survey. Participants were informed that the survey would take about 10 minutes to complete. The finishing of the survey was viewed as consent. The study was approved by Cornell University Institutional Review Board for Human Participant Research with the Protocol ID number 2003009492. The survey questionnaire followed the checklist for reporting results of internet e-surveys (CHERRIES) [10], available as a supporting document.

### Survey and procedures

A questionnaire was developed based on previous studies that investigated risk perception and actions to reduce anxiety during SARS, N1H1, and the current COVID-19 pandemic [11, 12]. Before launching the survey through Qualtrics to recruit participants, the questionnaire was sent to three experts in survey design as well as a group of 20 graduate students in the applied economics area for reliability and validity check. The survey was then modified based on their comments and suggestions. The survey included the following questions which were presented to participants in order:

#### 1) Social demographic information

The survey elicited information about gender, age, race, education, and income level of respondents.

#### 2) Level of concern of getting infected by COVID-19 while shopping for groceries and other activities

The psychometric variables employed to measure perceived risk constructed in this study follow the early studies by Slovic [13] and Loewenstein [14], and more recent studies by Siegrist [15] and Sokolowska and Zaleskiewicz [16]. To elicit the perceived risk of shopping for groceries and other activities (e.g., staying at home, going for a walk and handling mail or packages) the survey asked respondents to rank risk perception of these activities on a Likert scale ranging from ‘not concerned at all’ (1) to ‘extremely concerned’ (5).

#### 3) Perceived benefits of wearing a facemask

The perceived benefits resulting from wearing facemasks were also evaluated. The survey asked respondents to state their level of agreement (from 1 ‘strongly disagree’, to 5 ‘strongly agree’) with three statements concerning the benefits of wearing facemasks: “wearing a facemask in a crowded place decreases my chance of getting infected by COVID-19”, “wearing a facemask reduces my tendency to touch my mouth, face, and eyes,” and “wearing a facemask largely protects me against smaller respiratory droplets.”

#### 4) Perceived risk when shopping for groceries before and after respondents were exposed to an information intervention

The information intervention is a preventive action taken by grocery stores, ensuring the use of masks and gloves by employees to influence shoppers’ perceived risk. The hypothesized change in behavior due to cognitive factors follows the study of Rogers [17].

Before being exposed to the information intervention, respondents were asked to report their level of agreement with two statements measuring the perceived risk of COVID-19 infection when shopping for groceries: “Store employees have a high risk of getting infected" and "Going to the grocery store puts me at a high risk of getting infected.”

Then, respondents were asked to consider a hypothetical situation in which, at the entrance of the grocery store, signage states, "To assure your safety and minimize direct contact, starting today, in addition to our routine sanitation, employees will wear facemasks and gloves." The rationale for employing this preventive action is that the use of facemasks has been controversial since the pandemic began [18, 19] with recent evidence suggesting that wearing facemasks is effective in reducing the risk of respiratory virus infection [20]. A few days after the survey was distributed, the CDC reversed its guidance on facemasks from not recommending their use for people who were not sick to urging all people to wear any type of facemask, even a makeshift. Furthermore, there seems to be no guidance of any sort on gloves. This action is easy to implement and can be an effective way to reduce perceived risks. After the information intervention, respondents were asked to evaluate two statements similar to the ones evaluated before the information intervention, regarding their perceived risks for themselves and for store employees while shopping.

#### 5) Other factors that potentially influence shoppers’ risk perception

We follow the theory of social amplification or risk to control and test variables that amplify risk and leads to behavioral changes [21]. Thus, the survey also asked respondents if they had family members working in grocery stores or in the health care system, whether family members have been infected by COVID-19, or are experiencing any underlying health conditions.

After the survey was closed on April 10, 2020, the data were downloaded from the Qualtrics site and were imported to STATA 16 (StataCorp, College Station, Texas 77845 USA) for analysis. Respondents who completed the survey in less than half the median of completion time (10 minutes) were screened out to avoid including respondents who did not take the survey seriously. Consequently, a total of 674 valid responses were collected.

At the start of this survey, the official recommendation was not to wear facemasks unless the individual presented COVID-19 symptoms. Healthy people were not recommended to wear facemasks at that time. On April 3, 2020, the second day we launched the survey, the US government changed this recommendation, advising all people, including healthy, to wear facemasks in public when social distancing was not possible. In the meantime, some grocery stores required all people (customers as well as employees) to wear facemasks while at the grocery store. The context of our analysis is a situation where the use of facemasks was not enforced neither at the grocery stores nor to their customers.

## Results

### Respondents

The respondents had the following demographic characteristics (Table 1). 61% were female, with a mean age of 41 years. 71% were White, 9% Asian, and 10% African American. Half of the participants reside in metropolitan areas. Nearly 70% of respondents were employed, and 37% of those who were not employed had been laid off or furloughed due to COVID-19. Children under 10 were present in 38% of households, and 19% had a family member older than 65 years. Education level varied among respondents with 30% holding a postgraduate degree, 24% and 31% completed an undergraduate or associate degree, respectively, and the rest had a high school degree.

**Table 1:** Summary statistics (n = 674).

**Perceived concern for various activities (scale 1–5)**		
Perceived concern for going grocery shopping (score 4–5, very-extremely concerned)	70%	
Perceived concern for going for a walk (score 4–5, very-extremely concerned)	45%	
Perceived concern for handling mail or packages (score 4–5, very-extremely concerned)	38%	
Perceived concern for staying at home (score 4–5, very-extremely concerned)	19%	
**Perceived risk of getting infected (score 1–5, from strongly disagree-strongly agree)**	Before information intervention	After information intervention
Perceived themselves to have a high risk of getting infected (mean score)	4.0	2.5
Perceived store employees to have a high risk of getting infected (mean score)	4.3	2.1
**Perceived benefits of wearing facemasks**		
The perceived benefit of facemasks for reducing the chances of getting infected **(**scale 4–5, agree-strongly agree)	69%	
The perceived benefit of facemasks for reducing the tendency to touch nose, eyes, and face (scale 4–5, agree-strongly agree)	77%	
The perceived benefit of facemasks for protecting against smaller respiratory droplets (scale 4–5, agree-strongly agree)	75%	
**Social demographic information**		
White	71%	
Asian	9%	
African Americans	10%	
Age (mean)	41	
Age (by group)	% of male	% of female
21–29	26%	74%
30–39	41%	59%
40–49	49%	51%
50–59	42%	58%
60+	42%	58%
Education level (scale 1–4, high school-postgraduate degree)	2.38	

The next step in the analysis is to confirm whether respondents are concerned about getting infected when shopping for groceries (Table 1). Approximately 70% of respondents felt very or extremely concerned while grocery shopping. As expected, this concern is high relative to other activities, such as handling mail and packages (38% reported to feel very or extremely concerned), going for a walk (45% reported to feel very or extremely concerned), and staying at home (19% reported to feel very or extremely concerned).

### Perceived risk for in-store grocery shopping and other activities

Given the high level of perceived risk, a relevant question is how to alleviate concerns regarding grocery shopping. To answer this question, this study investigated how and to what extent the information intervention impacts shoppers’ perceived risk for themselves and store employees. Before presenting the information treatment, participants’ average risk perception rating for themselves and for store employees was 4.0 and 4.3, respectively (scores ranging from 1 to 5, with 1 being the low risk level and 5 being the high risk level). Participants’ risk perception ratings were significantly lowered after the information intervention. Specifically, after the information intervention, participants’ average risk perception rating for themselves and for store employees reduced to 2.5 and 2.1, respectively. As a result, the information intervention significantly reduced participants’ average risk perception for themselves by 37.5% and for store employees by 51.2%.

Regarding perceived benefits of wearing facemasks, 69%, 77%, and 75% of respondents agreed or strongly agreed that wearing facemasks reduces their chance of getting infected, reduces their tendency to touch face, nose, and eyes; and protects them from smaller respiratory droplets, respectively (Table 1).

### Effect of information treatment on perceived risk

While a comparison based on descriptive statistics is informative, to assess the impact of the information treatment, it is critical to control for other factors that may affect risk perceptions. Consequently, two multivariate statistical models were specified to estimate the effect of the information treatment on perceived infection risks of grocery shoppers regarding themselves and the store employees.

#### Dependent variables

The first dependent variable (*Riskemployee*) measures perceived risk for store employees getting infected with COVID-19. It has five levels ranging from 1 to 5, with 1 being lowest risk perception and 5 being the highest risk perception. The second dependent variable (*Riskself*) measures respondents’ perceived risk of themselves getting infected with COVID-19. Similar to *Riskemployee*, it has five levels, with 1 being the lowest risk perception and 5 being the highest perception.

#### Predictor variables

The critical research question is the influence of the information intervention on perceived risk of contagion. Therefore, a binary variable (*infodu*) was created and coded as one if the responses were collected after the information intervention, and as zero if the responses were collected before the information intervention.

In addition, three predictor variables were generated to represent the perceived benefits of wearing facemasks. The variable *mask_reducechance* equals one if respondents reported to agree or strongly agree that using a mask reduces the chance of contagion, zero otherwise. The variable *mask_reducetendency* equals one if respondents reported to agree or strongly agree that using a mask reduces the tendency to touch one’s face, nose, and eyes. The variable *mask_protect* equals one if respondents reported to agree or strongly agree that using a mask protects against smaller respiratory droplets.

The model also controlled for other predictor variables that may influence shoppers’ risk perception. Specifically, the variable *concernlevel* measured the level of concerns that respondents reported regarding grocery shopping. The variable *contagiouslevel* measured shoppers’ perceived contagion level of COVID-19. Respondents reported their levels of agreement to a statement described as “COVID-19 is highly contagious” from strongly disagree (1) to strongly agree (5). In addition, the following predictor variables were generated and included in the model 1) *hhdoctor* equals one if respondents have family members who work in the health care system, zero otherwise; 2) *hhshopwork* equals one if respondents have family members who work in food retail stores, zero otherwise; 3) *hhinfected* equals one if respondents have family members who are infected by COVID-19, zero otherwise; 4) *undercondition* equals one if respondents have an underlying health condition, zero otherwise. To control the potential impact of CDC revising its facemask use guideline on shoppers’ risk perception, a variable *date* is generated and is coded as one if the responses were collected after CDC revised its facemask guideline on April 3^rd^, 2020, zero if collected before CDC revised its guideline. Finally, the multivariate model controls for demographic characteristics of respondents, including *female* (equals one if female, zero otherwise), *Asian* (equals one if Asian, zero otherwise), *education* (self-reported level), and *age* (self-reported).

Because each dependent variable has five levels, two panel-data ordered logistic regressions were employed to estimate the effect of information (*infodu*) on perceived risk, controlling for the predictors explained above. The empirical model specifications were included in the S1 Appendix. The ordered logistic regression results for the critical predictor variables are presented in Table 2 (see S1 and S2 Tables in S2 Appendix for the full regression results). Columns 2 and 3 show the coefficients of the predictor variables for models with *Riskself* as the dependent variable (model 1) and *Riskemployee* as the dependent variable (model 2), respectively. To further test whether the change in risk perception depends on shoppers’ behavior in facemask use, we created a dummy variable *facemaskuse* that equals to one if shoppers wore any type of facemasks while shopping at the time of the survey, zero otherwise. At the time of data collection, facemask use was not mandatory by the government. We hence created an interaction term facemask*infodu and included them in the analysis. Columns 4 and 5 show the coefficients of the predictor variables for models with *Riskself* as the dependent variable (model 3) and *Riskemployee* as the dependent variable (model 4), respectively, while including the *facemaskuse* variable and the interaction term in the regression.

**Table 2:** Panel data ordered logistic regression results of risk perception.

	Model 1	Model 2	Model 3	Model 4
VARIABLES	Perceived risk for themselves (*Riskself*)	Perceived risk for employees (*Riskemployee*)	Perceived risk for themselves (*Riskself*)	Perceived risk for employees (*Riskemployee*)
*infodu*	-2.517***	-3.951***	-1.663***	-3.573***
	(0.120)	(0.152)	(0.162)	(0.191)
*date*	0.233	-0.020	0.220	-0.025
	(0.188)	(0.188)	(0.188)	(0.189)
*facemaskuse*			0.811***	0.355**
			(0.155)	(0.162)
facemask*infodu			-1.562***	-0.663***
			(0.205)	(0.209)
*concernlevel*	0.425***	0.070	0.438***	0.067
	(0.055)	(0.056)	(0.056)	(0.057)
*contagiouslevel*	0.125	0.212***	0.118	0.208**
	(0.081)	(0.082)	(0.081)	(0.082)
*mask_reducechance*	-0.120*	-0.104*	-0.123*	-0.107*
	(0.062)	(0.063)	(0.063)	(0.063)
*mask_reducetendency*	-0.095*	-0.097*	-0.096*	-0.098*
	(0.053)	(0.055)	(0.053)	(0.055)
*mask_protect*	-0.057	-0.190***	-0.070	-0.192***
	(0.068)	(0.070)	(0.068)	(0.070)
*hhdoctor*	-0.109	-0.240*	-0.125	-0.243*
	(0.133)	(0.136)	(0.134)	(0.137)
*hhshopwork*	0.229	0.339**	0.239*	0.337**
	(0.142)	(0.147)	(0.142)	(0.147)
*age*	-0.006	-0.008*	-0.006*	-0.007*
	(0.004)	(0.004)	(0.004)	(0.004)
*education*	-0.039	-0.085	-0.043	-0.086*
	(0.051)	(0.052)	(0.051)	(0.052)
Number of id	673	673	673	673
Wald chi^2^	484.80	692.93	525.29	698.90
Prob>chi^2^	0.000	0.000	0.000	0.000
Observations	1,346	1,346	1,346	1,346

Robust standard errors in parentheses

*** p<0.01

** p<0.05

* p<0.1

Column 2 of Table 2 shows that the coefficient of *infodu* is negative and statistically significant (1% level), indicating that respondents’ perception of being at a high risk of getting infected while shopping for groceries decreased after the information intervention. The odds ratio for variable *infodu* is 0.081, indicating that, after the information intervention, the odds for shoppers perceiving themselves to have a high risk is 0.081 times lower than before the information intervention (see S1 Table in S2 Appendix for full regression results). That is, the information intervention made people feel at a substantially lower risk of getting infected by COVID-19. Regarding other explanatory variables, the positive and statistically significant coefficient of *concernlevel* indicates that the more concerned respondents were about going grocery shopping, the more likely they perceived themselves as being at a high risk of getting infected by COVID-19. The coefficient of *contagiouslevel* is positive and significant (1% level), suggesting that the more contagious respondents think COVID-19 is, the more likely they perceived themselves to be at a high risk of getting infected. The coefficients for *mask_reducechance* and *mask_reducetendency* are negative and significant, indicating that the respondents who perceived more benefits of wearing facemasks were less likely to perceive themselves as being at a high risk of getting infected. The coefficient of the *date* variable is not significant here. That is, no evidence suggests a significant impact of change in the government messages about facemask use during data collection on shoppers’ risk perception for themselves. Other control variables in the model exhibit the expected signs but are not statistically significant at the 5% level.

Results in Column 3 of Table 2 indicate that the coefficient of *infodu* is negative and statistically significant (at the 1% level), suggesting that, after the information intervention, respondents’ perception of employees having a high risk of getting infected significantly reduced compared with before the information intervention. The odds ratio of *infodu* is 0.019 in model 2, indicating that, after the information intervention, the odds for shoppers perceiving employees to have a high risk is 0.019 times lower than before the information intervention (see S1 Table in S2 Appendix for full regression results). Regarding other predictor variables, the coefficient of *contagiouslevel* is positive and significant, suggesting that the more contagious respondents think COVID-19 is, the more likely they perceived store employees to be at a high risk of getting infected. The coefficient for *mask_reducechance*, *mask mask_reducetendency and mask_protect* are negative and significant, indicating that the respondents who perceive more benefits of wearing facemasks, the less likely they perceive employees as being at a high risk of getting infected. Regarding other control variables, shoppers who had family members working in grocery stores were more likely to perceive store employees as being at a high risk of getting infected, whereas, shoppers who had family members working in the health care system are less likely to perceive store employees as being at a high risk of getting infected. Older participants are less likely to perceive store employees as being at a high risk of getting infected than their younger counterparts. The coefficient of the *date* variable is not significant here. That is, no evidence suggests a significant impact of change in the government messages about facemasks during data collection on shoppers’ risk perception for store employees. Other control variables in the model exhibit the expected signs but are not statistically significant at the 5% level (see S2 Appendix for full regression results).

Columns 4 and 5 of Table 2 present the model with *Riskself* and *Riskemployee* as dependent variables, respectively, while including interaction term between the use of facemasks and the information intervention. The results in Column 4 show that the coefficient of *infodu*, representing the difference in perceived risk before and after information for shoppers who did not wear facemasks (*facemaskuse* = 0), is negative and significant. This suggests that the perceived risk for shoppers who did not wear facemasks is significantly lower after the information intervention. In addition, for shoppers who wore facemasks (*facemaskuse* = 1), the difference in perceived risk before (when *facemaskuse* = 1) and after information (when *facemaskuse* = 1, *infodu* = 1 and facemask*infodu = 1) is represented by the sum of the coefficient of *infodu* (C_*infodu*_ = -1.663) and the coefficient of facemask*infodu (C_facemask*infodu_ = -1.562) which is negative and statistically significant (-1.663+ (-1.562)). This suggests that shoppers who wore facemasks perceived a much lower risk after information treatment. The magnitude of the risk reduction resulting from the information intervention is greater for shoppers who wore facemasks than those who did not wear facemasks. That is, the coefficient of facemask*infodu (C_facemask*infodu_ = -1.562) is negative and significant implies that the information effect on perceived risk reduction for people who wore facemasks (*facemaskuse* = 1) is greater than the effect for people who did not wear facemasks (*facemaskuse* = 0). Therefore, the information intervention is more effective for shoppers who wore facemasks than those who did not wear facemasks.

Similarly, the results in column 5 shows a negative and significant coefficient of the interaction term (C_facemask*infodu_ = -0.663) indicating that the information treatment effect on perceived risk reduction of store employees getting infected for shoppers who wore facemasks (*facemaskuse* = 1) is greater than the effect for shoppers who did not wear facemasks (*facemaskuse* = 0). That is, the magnitude of the perceived risk reduction of store employees getting infected for shoppers who wore facemasks is greater than those who did not wear facemasks. These results make sense. The possible reason is that at the beginning of the pandemic when the survey data was collected, facemask use was not even recommended let alone required, people who wore them voluntarily may be more cautious (or more risk averse) and take COVID-19 more seriously. They may perceive the information on grocery stores requiring employees to wear facemasks as a very proactive action to keep shoppers and employers safe.

## Discussion

Previous studies highlight the importance of examining risk perception for effective pandemic management (e.g., SARS, H1N1) [8, 22, 23]. However, few studies have investigated this issue in the context of COVID-19, notably people’s risk perception regarding grocery shopping, as well as effective strategies to reduce risk perception. To fill this gap, this study investigated the level of perceived risk when shopping for groceries in brick-and-mortar stores relative to other activities during COVID-19 pandemic in the US. It also examined the impact on perceived risk via a preventive action on the mandatory use of masks and gloves by store employees. An online survey was conducted to identify and question grocery shoppers in NYS and WA. Results indicate that people were much more concerned about grocery shopping during the pandemic relative to other activities, such as going for a walk or handling mail/packages, which is in line with previous literature [24].

However, perceived risk of being infected by COVID-19 was significantly reduced by 37.5% after considering an information intervention, namely a proactive action by grocery stores requiring the use of masks and gloves by employees. The results also showed that shoppers perceived store employees to be at a high risk of getting infected with COVID-19. Respondents’ perception of store employees being at a high risk of getting infected by COVID-19 significantly decreased by 51.2% after the information intervention. The study further showed that the information intervention are more effective for shoppers who wore facemasks voluntarily at the beginning of the pandemic than those who did not.

## Conclusion

Our findings suggest that at the beginning of the pandemic, before facemask wearing became mandatory, supermarkets requiring employees to wear facemask and gloves as preventive actions could significantly reduce shoppers’ concerns and anxiety about the risk of contagion while protecting their employees. At that time, the official stance of the CDC on facemask wearing was that it did not recommend its use for protection against COVID-19 infection, and the Surgeon General advised the public to stop buying face masks [18]. Despite the official stance on public facemask wearing, consumers felt safer when store employees wear facemasks. One year into the pandemic, facemask wearing is mandatory and people were informed about its benefits. This study revealed the need for the continuation of the proactive actions taken by food retailers during the pandemic.

The findings are relevant to other situations where a six-feet (about 1.83 meters) social distance may be difficult to maintain and customers may perceive a high risk of getting infected with COVID-19 such as salons, gyms, banks and shopping malls,. These proactive actions are ultimately essential to reduce people’s anxiety due to the pandemic and the unwitting transmission of the virus from asymptomatic customers [25–27], in preparation for the reopening of the economy, and the ongoing second/third wave of COVID-19. On the other hand, customers may be more likely to patronize businesses that implement preventive measures aimed at reducing customers’ risk perceptions and anxiety. Even in a new state of the world where safety measures are required by regulations, taking further actions to reduce customers’ risk perceptions may be preferred by customers. According to Yeung and Morris [28], customers develop strategies to minimize risk exposure. Some of these strategies include stopping or reducing a purchase, or look for alternatives [29]. Risk perception of consumers may affect their selection of stores, while proactive measures of a store may improve the image of the store and positioning among customers [30].

As the number of vaccines being distributed increases, along with increasing pandemic fatigue, people may be more reluctant to comply with safety measures, despite the ongoing risk of infection. Governments and institutions should encourage people to observe the safety recommendations and to continue informing about the current and potential risks of non-compliance especially when the costs of complying with some of these measures, like facemask wearing, is low. Based on our results, facemask wearing, despite the arguments against by some sectors of society, would decrease people’s anxiety of COVID-19 infection.

### Limitations

The study has several limitations. First, the data were collected at the beginning of the pandemic, but shoppers’ risk perception may change over time. The perceived risk perceptions measured in this study may not be generalized to other stages of the pandemic. Second, the risk perceptions measured may not be generalized to other US regions. The data were collected in Washington and New York, the two states with the highest number of confirmed cases at the beginning of the pandemic. Shoppers in other states may not share the same risk perceptions as those in Washington and New York.

With the development the pandemic in the US, future research could explore and compare how shoppers risk perception changed over time. One fertile area of research is to examine how and to what extent the mandatory facemasks policy impacts shoppers’ risk perception and their attitude toward the pandemic. Finally, future research could also explore whether possible ‘pandemic fatigue’ influences shopper risk perceptions and subsequent social behaviors.

## Supporting information

S1 Appendix (DOCX)

S2 Appendix (DOCX)

S1 Data (XLS)
