# Correction: Strategies to reduce COVID-19 risk perception among grocery shoppers in the US: A survey study

**DOI:** 10.1371/journal.pone.0256191

**Published:** 2021-08-10

**Authors:** Jie Li, Leslie J. Verteramo Chiu, Miguel I. Gómez, Nelson L. Bills

The word "COVID-19" is missing from the article title. The correct title is: Strategies to reduce COVID-19 risk perception among grocery shoppers in the US: A survey study. The correct citation is: Li J, Verteramo Chiu LJ, Gómez MI, Bills NL (2021) Strategies to reduce COVID-19 risk perception among grocery shoppers in the US: A survey study. PLoS ONE 16(4): e0251060. https://doi.org/10.1371/journal.pone.0251060
