# Supplementary material for: Strategies to reduce risk perception among grocery shoppers in the US: A survey study
Source: PLoS One. 2021 Apr 28;16(4):e0251060. doi: 10.1371/journal.pone.0251060 (PMC8081199; doi:10.1371/journal.pone.0251060)
Supplement: S1 Appendix — (DOCX) [file pone.0251060.s001.docx]

S1 Appendix.

For the ordered logit model using *Riskself* five-level ratings as the dependent variable, the econometrics equations are represented in the following:

$$\left( 1 \right) Riskslef=1 if {Riskself}^{*}\leq j_{1}$$

$$\left( 2 \right) Riskslef=2 if j_{1}\leq{Riskself}^{*}\leq j_{2}$$

$$\left( 3 \right) Riskslef=3 if j_{2}\leq{Riskself}^{*}\leq j_{3}$$

$$\left( 4 \right) Riskslef=4 if j_{3}\leq{Riskself}^{*}\leq j_{4}$$

$$\left( 5 \right) Riskslef=5 if {Riskself}^{*}\geq j_{4}$$

Where ${Riskself}^{*}$ is a continuous, unmeasured latent variable which has various threshold points indicated by $j_{i}, i=1\ldots.4$.

$$\left( 6 \right) {Riskself}^{*}=\sum_{k=1}^{K} \beta_{k}X_{k}+\varepsilon_{riskself}=Z+\varepsilon_{riskself}$$

$$\left( 7 \right) Z=\beta_{1}infodu+\beta_{2}concernlevel+\beta_{3}contagiouslevel+\beta_{4}maskprotect+\beta_{5}maskreducechance+\beta_{6}maskreducetendency+\beta_{7}hhdoctor+\beta_{8}hhshopwork+\beta_{9}hhinfected+\beta_{10}undercondition+\beta_{11}Female+\beta_{12}Age+\beta_{13}Education+\beta_{14}Asian+\beta_{15}date$$

Where $\varepsilon_{riskself}$is a random disturbance term, which has a standard logistic distribution. In our study, *Riskself* takes five levels from 1 to 5. So the estimation equations can be simplified as:

$$\left( 8 \right) P\left( Riskself=1 \right)=\frac{1}{1+exp(Z-j_{1})}$$

$$\left( 9 \right) P\left( Riskself=2 \right)=\frac{1}{1+exp(Z-j_{2})}-\frac{1}{1+exp(Z-j_{1})}$$

$$\left( 10 \right) P\left( Riskself=3 \right)=\frac{1}{1+exp(Z-j_{3})}-\frac{1}{1+exp(Z-j_{2})}$$

$$\left( 11 \right) P\left( Riskself=4 \right)=\frac{1}{1+exp(Z-j_{4})}-\frac{1}{1+exp(Z-j_{3})}$$

$$\left( 12 \right) P\left( Riskself=5 \right)=\frac{1}{1+exp(Z-j_{4})}$$

Similarly, for the ordered logit model using *Riskemployee* five-level ratings as the dependent variable, the econometrics equations are represented in the following:

$$\left( 13 \right) Riskemployee=1 if {Riskemployee}^{*}\leq\gamma_{1}$$

$$\left( 14 \right) Riskemployee=2 if \gamma_{1}\leq{Riskemployee}^{*}\leq\gamma_{2}$$

$$\left( 15 \right) Riskemployee=3 if \gamma_{2}\leq{Riskemployee}^{*}\leq\gamma_{3}$$

$$\left( 16 \right) Riskemployee=4 if \gamma_{3}\leq{Riskemployee}^{*}\leq\gamma_{4}$$

$$\left( 17 \right) Riskemployee=5 if {Riskemployee}^{*}\geq\gamma_{4}$$

Where ${Riskemployee}^{*}$ is a continuous, unmeasured latent variable which has various threshold points indicated by $\gamma_{i}, i=1\ldots.4$.

$${\left( 18 \right) Riskemployee}^{*}=\sum_{k=1}^{K} \alpha_{k}X_{k}+\varepsilon_{employee}=T+\varepsilon_{employee}$$

$$\left( 19 \right) T=\alpha_{1}infodu+\alpha_{2}concernlevel+\alpha_{3}contagiouslevel+\alpha_{4}maskprotect+\alpha_{5}maskreducechance+\alpha_{6}maskreducetendency+\alpha_{7}hhdoctor+\alpha_{8}hhshopwork+\alpha_{9}hhinfected+\alpha_{10}undercondition+\alpha_{11}Female+\alpha_{12}Age+\alpha_{13}Education+\alpha_{14}Asian+\alpha_{15}date$$

Where $\varepsilon_{employee}$is a random disturbance term, which has a standard logistic distribution. In our study, *Riskemployee* takes for five levels from 1 to 5. The estimation equations can be simplified as:

$$\left( 20 \right) P\left( Riskemployee=1 \right)=\frac{1}{1+exp(T-\gamma_{1})}$$

$$\left( 21 \right) P\left( Riskemployee=2 \right)=\frac{1}{1+exp(T-\gamma_{2})}-\frac{1}{1+exp(T-\gamma_{1})}$$

$$\left( 22 \right) P\left( Riskemployee=3 \right)=\frac{1}{1+exp(T-\gamma_{3})}-\frac{1}{1+exp(T-\gamma_{2})}$$

$$\left( 23 \right) P\left( Riskemployee=4 \right)=\frac{1}{1+exp(T-\gamma_{4})}-\frac{1}{1+exp(T-\gamma_{3})}$$

$$\left( 24 \right) P\left( Riskemployee=5 \right)=\frac{1}{1+exp(T-\gamma_{4})}$$
