# Supplementary material for: Strategies to reduce risk perception among grocery shoppers in the US: A survey study
Source: PLoS One. 2021 Apr 28;16(4):e0251060. doi: 10.1371/journal.pone.0251060 (PMC8081199; doi:10.1371/journal.pone.0251060)
Supplement: S2 Appendix — (DOCX) [file pone.0251060.s002.docx]

S2 Appendix.

S1 Table. Full regression results for Model 1 and Model 2 with Odds ratio

|  | Model 1 |  | Model 2 |  |
| --- | --- | --- | --- | --- |
| VARIABLES | Perceived risk for themselves | Odds ratio | Perceived risk for employees | Odds ratio |
|  |  |  |  |  |
| *infodu* | -2.517*** | 0.081 | -3.951*** | 0.019 |
|  | (0.120) |  | (0.152) |  |
| *date* | 0.233 | 1.262 | -0.0202 | 0.980 |
|  | (0.188) |  | (0.188) |  |
| *concernlevel* | 0.425*** | 1.530 | 0.0696 | 1.072 |
|  | (0.0551) |  | (0.0560) |  |
| *contagiouslevel* | 0.125 | 1.134 | 0.212*** | 1.236 |
|  | (0.0812) |  | (0.0820) |  |
| *mask_reducechance* | -0.120* | 0.887 | -0.104* | 0.901 |
|  | (0.0618) |  | (0.0626) |  |
| *mask_reducetendency* | -0.0950* | 0.909 | -0.0970* | 0.908 |
|  | (0.0531) |  | (0.0548) |  |
| *mask_protect* | -0.0566 | 0.945 | -0.190*** | 0.827 |
|  | (0.0675) |  | (0.0699) |  |
| *hhdoctor* | -0.109 | 0.897 | -0.240* | 0.787 |
|  | (0.133) |  | (0.136) |  |
| *hhshopwork* | 0.229 | 1.257 | 0.339** | 1.403 |
|  | (0.142) |  | (0.147) |  |
| *Asian* | 0.0320 | 1.033 | 0.277 | 1.319 |
|  | (0.184) |  | (0.193) |  |
| *undercondition* | 0.00816 | 1.008 | 0.0944 | 1.099 |
|  | (0.108) |  | (0.111) |  |
| *hhinfected* | 0.104 | 1.110 | 0.131 | 1.140 |
|  | (0.149) |  | (0.152) |  |
| *female* | 0.141 | 1.151 | 0.124 | 1.133 |
|  | (0.110) |  | (0.113) |  |
| *age* | -0.00563 | 0.994 | -0.00761* | 0.992 |
|  | (0.00378) |  | (0.00394) |  |
| *education* | -0.0387 | 0.962 | -0.0846 | 0.919 |
|  | (0.0508) |  | (0.0522) |  |
|  |  |  |  |  |
| Observations | 1,346 |  | 1,346 |  |

Robust standard errors in parentheses

*** p<0.01, ** p<0.05, * p<0.1

S2 Table. Full regression results for Model 3 and Model 4 with Odds ratio

|  | Model 3 | Model 4 | |  |
| --- | --- | --- | --- | --- |
| VARIABLES | Perceived risk for themselves | | Perceived risk for employees | |
|  |  |  | |  |
| *infodu* | -1.663*** | -3.573*** | |  |
|  | (0.162) | | (0.191) | |
| *date* | 0.220 | | -0.0254 | |
|  | (0.188) | (0.189) | |  |
| *facemaskuse* | 0.811*** | 0.355** | |  |
|  | (0.155) | (0.162) | |  |
| *facemask*infodu* | -1.562*** | -0.663*** | |  |
|  | (0.205) | (0.209) | |  |
| *concernlevel* | 0.438*** | 0.0674 | |  |
|  | (0.0561) | (0.0571) | |  |
| *contagiouslevel* | 0.118 | 0.208** | |  |
|  | (0.0812) | (0.0824) | |  |
| *mask_reducechance* | -0.123* | -0.107* | |  |
|  | (0.0628) | (0.0634) | |  |
| *mask_reducetendency* | -0.0964* | -0.0981* | |  |
|  | (0.0532) | (0.0550) | |  |
| *mask_protect* | -0.0702 | -0.192*** | |  |
|  | (0.0678) | (0.0698) | |  |
| *hhdoctor* | -0.125 | -0.243* | |  |
|  | (0.134) | (0.137) | |  |
| *hhshopwork* | 0.239* | 0.337** | |  |
|  | (0.142) | (0.147) | |  |
| *Asian* | 0.0512 | 0.282 | |  |
|  | (0.186) | (0.193) | |  |
| *undercondition* | 0.0141 | 0.0942 | |  |
|  | (0.110) | (0.112) | |  |
| *hhinfected* | 0.102 | 0.131 | |  |
|  | (0.150) | (0.153) | |  |
| *female* | 0.162 | 0.126 | |  |
|  | (0.112) | (0.114) | |  |
| *age* | -0.00638* | -0.00743* | |  |
|  | (0.00382) | (0.00395) | |  |
| *education* | -0.0426 | -0.0859* | |  |
|  | (0.0510) | (0.0522) | |  |
|  |  |  | |  |
| Observations | 1,346 | 1,346 | |  |

Robust standard errors in parentheses

*** p<0.01, ** p<0.05, * p<0.1
